# Comparison between Physical Activity and Stress-Related Lifestyle between Orthorexic and Non-Orthorexic University Students: A Case–Control Study

**DOI:** 10.3390/nu16091340

**Published:** 2024-04-29

**Authors:** Sara Guidotti, Alice Fiduccia, Michele Murgolo, Carlo Pruneti

**Affiliations:** Clinical Psychology, Clinical Psychophysiology and Clinical Neuropsychology Laboratory, Department of Medicine and Surgery, University of Parma, 43126 Parma, Italy; alice.fiduccia@live.it (A.F.); michele.murgolo@studenti.unipr.it (M.M.); carlo.pruneti@unipr.it (C.P.)

**Keywords:** orthorexia, eating disorders, obsessive–compulsive disorders, lifestyle, physical exercise, physical activity, university students, mental health, stress, somatizations

## Abstract

(1) Background: The literature regarding orthorexia nervosa (ON) has well documented the association with other mental disorders, such as obsessive–compulsive and eating disorders. However, the research has not taken into account stress-related behavior and the conduction of physical activity (PA), both structured and unstructured. (2) Methods: In this cross-sectional study, 165 students of the University of Parma (92 females and 74 males) aged between 18 and 49 years old (mean = 24.62 ± 4.81) were consecutively recruited. The ORTO-15 questionnaire was used to divide the total sample into a group without orthorexia (score > 40) and a group with orthorexia (score < 40). All subjects completed the P Stress Questionnaire, and specific items were extrapolated from the Eating Habits Structured Interview (EHSI) to investigate lifestyle, including structured and unstructured PA. (3) Results: Subjects with orthorexia represented 83% of the total sample and reported higher levels of stress-related risk behaviors (i.e., sense of responsibility (t = −1.99, *p* = 0.02), precision (t = −1.99, *p* = 0.03), stress disorders (t = −1.38, *p* = 0.05), reduced spare time (t = −1.97, *p* = 0.03), and hyperactivity (t = −1.68, *p* = 0.04)) and a higher frequency of PA (i.e., hours spent training in structured PA, daily (t = −1.68, *p* = 0.05), weekly (t = −1.91, *p* = 0.03), and monthly (t = −1.91, *p* = 0.03), the tendency to carry out physical exercise even if tired (t = −1.97, *p* = 0.02), and to adhere to unstructured PA (i.e., moving on foot or by bike rather than using transport (t = 1.27, *p* = 0.04)). (4) Conclusions: The results confirmed the presence of hyperactivity at a motor and behavioral level in people with orthorexia. Further studies are necessary to highlight the causality between ON, stress, and physical activity but it may be possible to hypothesize that “obsessive” physical exercise may not generate the benefits generally known by the literature.

## 1. Introduction

Orthorexia is a neologism coined in Greek that translates to right appetite [1]. For individuals suffering from ON, their food must be natural and healthy [2]. The search for extremely pure food can lead to excessive attention to food with harmful and counterproductive consequences on psychophysical health, very similar to eating disorders (EDs) [3]. The term orthorexia nervosa (ON) refers to self-imposed dietary rules aimed at promoting health. The main symptom is an obsessive and insecure focus on eating foods considered healthy [4]. Although this disorder has not yet been included in diagnostic manuals, ON has already entered the vocabulary of clinical professionals and has come to the attention of researchers. Firstly illustrated by Steven Bratman in 1997 [5], this psychopathological condition was recently described in a 2019 review by Cena and colleagues [6], who highlighted two main possibly pathognomonic criteria: (1) obsessive attention (i.e., rigid dietary rules, recurrent and persistent worries related to food, and compulsive behaviors) on dietary practices believed to promote optimal well-being; and (2) clinically significant impairment (i.e., medical or psychological complications, great distress, and/or impairment of important areas of functioning) [7]. As regards eating behavior, other authors identified different types of healthy food [8]. Sometimes, healthy, correct, and safe characteristics of food emerge, while other times, the definition of food considers aspects mainly linked to food production, such as organic or biologically pure. In other cases, the definition of healthy food seems not to refer so much to biological quality but more to pseudo-moral aspects. In general, the diet followed by subjects with ON is characterized by a restrictive pattern in which the distortion of eating habits and the avoidance of specific foods can lead to a deficiency in essential nutrients or malnutrition and underweight. Emotional and behavioral alterations may be observed in terms of changes in eating habits, which may become ritualized and/or strictly controlled [9].

Research has recently enriched the ON literature, supporting the idea that it is not simply an altered eating habit but, rather, a disorder that falls within ED or obsessive–compulsive disorders (OCD), or that it even may represent a disorder in its own right [10].

In agreement with authors who emphasize the association with EDs, ON could be an emerging ED in itself or a possible premorbid symptom of other EDs [11]. The aspects that ON and ED would share would be (1) excessive rumination on food and, in particular, on specific aspects, such as preparation, consumption, and quality; (2) spending excessive time searching for and purchasing food; (3) strict food restrictions that can lead to a monotonous or insufficient diet; (4) concern about the negative consequences of eating specific foods; and (5) meal planning [6,7]. On the contrary, ON differs from ED due to some specific characteristics, such as (1) the primary concern for the quality of food (i.e., people with orthorexia are more concerned about the purity and wholesomeness of food than about the quantity); (2) an absence of compensatory behaviors (i.e., people with ON generally do not use compensatory behaviors, such as self-induced vomiting or the use of laxatives); and (3) greater attention to physical health (i.e., the focus is on physical health and not on body image) [6,7].

On the other hand, obsession, fixation, and worry are keywords often cited in ON studies. While obsession indicates a persistent and disturbing thought, fixation is a stereotyped behavior linked to an obsessive and unhealthy worry. In turn, worry refers to a state of discomfort mixed with interest, uncertainty, and apprehension, which takes on the concept of concern if characterized by a higher degree of alarm [6,12].

Nevertheless, psychiatric comorbidities seem to be frequent in ON. Recent studies have attested that depression and anxiety positively correlate with ON [13] as well as stress [14] and that all these factors can play the role of moderators on specific predictors (i.e., attachment) of ON [15]. In summary, several studies agree on the fact that when a healthy diet turns into ON, it leads to significant impairments and stress [16]. Although the comorbidities and the associations of different factors were described several times by researchers, a description of the association between ON and stress-related lifestyle, including PA, seems to be lacking. Furthermore, considering the high prevalence of ON in the specific category of university students (the data, variable depending on the instrument used, fluctuates between 3.3% [17] and 6.9% [18] and up to 80% [19] if detected with more sensitive instruments), it is essential to investigate the associations with behavioral factors that may aggravate mental health. For instance, ON symptoms appear to be related to the levels of physical exercise performed [20]. A theoretical model of the connections between ON and PA in the general population was tested via a structural equation model by Zohar and colleagues [21], in which the profile of participants with high levels of ON was associated with various psychological aspects (i.e., body dissatisfaction, difficulties in emotional regulation) as well as compulsive exercise. In a 2014 study, Lichtenstein and colleagues [22] documented the coexistence of exercise addiction with eating disorders and similar conditions. Nevertheless, previous studies found that 8.5% of Italian students were at risk of addiction to physical exercise [23]. In this regard, ON was strongly associated with the desire to improve physical and mental health, specifically among universities, at the cost of following a rigid program even in the face of injuries and illnesses [20]. Additionally, a couple of studies suggested that there may be an association between ON and frequency of physical activity, measured in hours of exercise per week, even among university students [24,25]. However, although a positive correlation was observed, it did not reach statistical significance.

In light of these assumptions, the aim that guided the present research was to investigate the conduction of PA and the presence of a stress-related lifestyle in a group of people with orthorexia, comparing them with a group of people without orthorexia. We hypothesize that people with ON may exercise excessively and adopt a stress-related lifestyle. These results could be consistent with the OCD and ED literature, as they would confirm the presence of motor–behavioral compulsions in association with the obsessions typical of EDs and OCD.

## 2. Materials and Methods

### 2.1. Participants

In this exploratory and cross-sectional study, 166 students (92 females and 73 males) aged between 18 and 49 years old were examined.

Criteria for inclusion in the study were age > 18 years old, completion of informed consent, and no history of psychiatric and/or neurological syndromes (e.g., previous head trauma, epilepsy, etc.) and/or physical diseases (i.e., sensory disturbances of sight and/or hearing) that may limit the administration of the tests.

### 2.2. Procedure

All of the participants consecutively went to the Laboratories of Clinical Psychology, Clinical Psychophysiology, and Clinical Neurophysiology of the Department of Medicine and Surgery of the University of Parma in the period between February and March 2024, responding to some posters that offered a link to book an in-person appointment via the Outlook calendar. For this reason, the sample was made up of students of various majors (i.e., medicine, psychology, educational sciences, communication, law, economics, nursing, biology, information, etc.). The researchers explained the purpose of the study and the instruments that would be administered to students without specifying the single scales so as not to nullify their face validity. Once the tests were administered, the participants were offered the option to book another appointment with a licensed clinical psychologist to possibly receive an exhaustive commentary and ask questions.

The Ethical Committee of the University of Parma approved the study (protocol number: 22186/2024). All the procedures were conducted following the Declaration of Helsinki and its later advancements in accordance with research involving human participants. Participants’ anonymity was preserved, and the data obtained were used solely for scientific purposes. All participants gave their consent to publish the results derived from their data.

### 2.3. Measures

To measure orthorexia, the most widespread scale translated into several languages was used, i.e., the Orthorexia Nervosa Questionnaire-15 (ORTO-15; Italian version) [26], which is made up of 15 items to be answered using a scale of 4-step Likert-style frequency (always, often, sometimes, never). The items reveal the presence of an obsessive habit in choosing, purchasing, preparing, and consuming foods that people consider healthy. The scores are calculated by adding the responses to each item, where a value of 1 corresponds to orthorexic characteristics, and a value of 4 corresponds to normal behaviors. The final test score can vary between 15 and 60, and the threshold value, below which diagnosis of ON can be made, is established at <40. Different studies have confirmed the substantial validity of the test for the cut-off (sensitivity 100%, specificity 73.6%, positive predictive value 73.6%, negative predictive value 0%) [27]. In the present study, a Cronbach’s alpha equal to 0.72 was obtained.

The P Stress Questionnaire (PSQ) [28] is a tool made up of 32 items grouped into six scales: sense of responsibility (SR), vigor (V), stress disorders (SD), precision and punctuality (PP), spare time (ST), and hyperactivity (H). It detects whether there is a present risk for stress-related physical disorders attributable to the presence of a stress-related lifestyle. In some cases, a stress-related lifestyle falls within a phase characterized by stress overload, whereas, in other cases, it is associated with specific personality configurations that predispose to stress-related disorders, such as “Type A Behavior”. The SR factor includes attitudes related to taking life and personal duties too seriously. This means that a person may continuously engage in work or other activities without taking breaks. The V factor includes items that refer to the feeling of having characteristics such as vitality, energy, and stress resistance. The SD factor consists of items linked to troubles and problems usually related to stress reactions, such as a lack of sexual interest and difficulty falling asleep. The PP factor includes behaviors characterized by spitefulness, precision, and punctuality. The ST factor is linked to items regarding the care of oneself and the capability and possibility of relaxing and taking breaks from work. The H factor refers to behaviors characterized by extreme activity and the presumption of being able to resist fatigue well. The Italian standardization reported values between 0.40 and 0.70 for each scale. The Cronbach’s alpha calculated including all scales was equal to 0.66. The Stanine scores had a distribution between 1 and 9, with a mean of 5 and a standard deviation of 1.96.

The assessment of PA included an extrapolation of the Eating Habits Structured Interview (EHSI) [29]. The EHSI is a comprehensive interview useful for the clinical context, as it consists of 5 sheets and 53 items and addresses data and medical history, lifestyle, PA, body perception, and eating habits. Consistent with the aim of this study, specific items of Sheet 2 were used to define the conduction of structured PA, the number of daily and weekly hours dedicated to it, and behaviors that describe the preference for carrying out unstructured PA compared to alternative activities. More specifically, the unstructured PA items are as follows: (1) “From 0 to 100, how often do you prefer to take the stairs rather than take the elevator?”; (2) “From 0 to 100, to travel a short distance, how often do you prefer to use a bike or walk rather than use a means of transport (e.g., bus or moped)”; (3) “When you have free time, from 0 to 100, how often do you prefer to go for a walk alone or in a company rather than do sedentary activities (i.e., reading, computer, TV)”; (4) “If a friend or acquaintance of yours lived not far from your home, how often would you prefer to go to him rather than call him?”; (5) “At the seaside, how often do you prefer to swim or play on the beach (e.g., beach volleyball, rackets) rather than lie down and sunbathe?”; (6) “After a day of studying, how often can you do physical activity even if you feel tired and listless?”; (7) “You have just argued with your partner. You feel upset and sad and would like to eat something. How many times, instead of eating, do you prefer to talk about what happened with someone or go for a walk?”. The response to conducting structured PA was coded as a dichotomous variable (yes/no), and the number of hours per day/week/month was a continuous variable. The responses to the unstructured PA items were considered continuous variables as well.

### 2.4. Statistical Analysis

All statistical analyses were performed using SPSS (Version 28.0.1.0; IBM Corp, Armonk, NY, USA). First, descriptive statistics were performed with the calculation of the mean (M) and standard deviation (SD). Next, tests for skewness and kurtosis and the Kolmogorov–Smirnov test were used to determine the normality of distribution. Then, in a multicollinearity test, no extreme coefficient values ≥0.8 were found among the independent variables, indicating a low risk of multicollinearity. All the independent variables had variance inflation factors ≤10 and tolerance ≥0.1, indicating the absence of multicollinearity. Lastly, since all the assumptions for the conduction of parametric statistics were respected, differences between groups in terms of the sociodemographic (i.e., age, gender, marital status, and education level) and the clinical characteristics (PSQ scores and EHSI extrapolated items about structured and unstructured PA) were calculated using the chi-square test and independent samples *t*-test.

## 3. Results

First, an ORTO-15 score of 40 was used as a cut-off to identify a group of university students with orthorexia. The group of participants with a score < 40 comprised 138 people, representing 83.13% of the total sample. Concerning the socio-demographic variables, the group of students with orthorexia did not differ from that composed of individuals without orthorexia for any of the variables observed (Table 1).

The comparison between the groups regarding the scores on the sub-scales of the PSQ revealed that the subjects with orthorexia had higher levels of sense of responsibility, stress disorder, and hyperactivity. Additionally, they were more precise and punctual and reported having difficulty taking advantage of free time, demonstrating a greater predisposition to carry out risky behaviors for stress-related physical disorders (Table 2).

Comparing the groups based on the characteristics of physical exercise, both structured and unstructured, it emerged that the people with orthorexia increased their weekly hours of PA, although they were involved in a sporting activity as much as the group without orthorexia. On top of this, they performed PA even if they were tired. Furthermore, individuals with orthorexia were more inclined to carry out unstructured PA (i.e., they preferred to move on foot or by bike rather than use means of transport) (Table 3).

## 4. Discussion

The research hypotheses that guided the present study were validated, as significant differences in lifestyle and in carrying out PA emerged by dividing the total sample into subjects with orthorexia and subjects without orthorexia. Specifically, it was hypothesized that the subjects with ON would adopt a stress-related lifestyle and perform greater levels of structured and unstructured PA. The two samples, created based on the discriminant power of the ORTO-15 cut-off (a score lower than 40 indicates orthorexia), differed significantly concerning lifestyle and PA. The subjects with ON tended to have stress-related lifestyles, as documented by the PSQ factors’ scores. A high sense of responsibility associated with motor hyperactivity, precision, and difficulty in benefiting from free time are typical characteristics of specific personality configurations in which obsessiveness is accompanied by compulsions [30,31], with the risk of developing stress-related physical disorders [32,33].

Based on these considerations, the increase in the practice of physical exercise substantiated by the comparison between the two groups in the items extrapolated from the EHSI is relevant. The analyses conducted underlined that the individuals with orthorexia dedicated more time daily, weekly, and monthly to performing physical exercise, although the hours of PA spent by the students with ON did not fall within the definition of an “addicted” exerciser (for instance, a “healthy” exerciser may complete five 30 min aerobic sessions per week—2.5 total hours—whereas an “addicted” one may complete five 3h aerobic sessions per week—15 total hours) [20]. Nonetheless, the ON subjects were more likely to carry out unstructured PA (i.e., going somewhere on foot or by bike rather than using a means of transport) and to train despite being tired. These aspects described by our research could highlight the obsessive aspects comorbid with OCD, characterized by highly disturbing, intrusive, and unwanted thoughts or obsessions, which the patient unsuccessfully tries to regulate by engaging in ritualized behaviors to defuse obsessions [9]. The propensity to carry out compulsive behaviors despite negative consequences is considered the main characteristic of OCD, which, for this reason, is conceptualized as a disorder of self-control and behavioral inhibition [8,34]. The reduced ability to inhibit intrusive cognitions and interrupting repetitive activity represent the impairment of inhibitory regulation typical of emotional and behavioral psychopathological disorders [12,35].

In light of these assumptions, it is possible to hypothesize that the obsessiveness of ON affects not only eating but also PA, manifesting in both contexts with difficulty in shifting between mental processes to generate adaptive behavioral responses. By way of illustration, neuroscience research suggested that cognitive flexibility (i.e., investigated through attention-shifting tasks) is impaired in individuals with obsessive–compulsive symptoms [36,37,38,39,40] due to the under-activation of prefrontal regions [38,39,40] as well as the limbic structures responsible for the emotional processing of stimuli [41,42,43,44,45].

By demonstrating an increase in the performance of structured and unstructured PA in subjects with orthorexia, our findings support theories according to which obsessive–compulsive spectrum disorders, including ON, are characterized by pathological cognitive processes capable of generating emotional arousal, which the individual is unable to manage or inhibit except by implementing motor behaviors. In other words, compulsions and motor behaviors are a way to facilitate the relaxation of somatic tension [46], and stress management may represent one of the motivations for exercise [20]. Thus, the behaviors implemented by people with obsessive–compulsive spectrum disorders (i.e., ON) would be among the strategies, albeit maladaptive, used to better manage the discomfort generated by thoughts [47]. Following this perspective, the high scores in the PSQ factors found in our study manifested high levels of behavioral activity as a lifestyle in the group of subjects with orthorexia.

Looking at our research, the analysis of the PSQ factors attested to a tendency towards hyper-responsibility and motor–behavioral hyperactivity associated with precision and difficulty in carving out some free time in the group of subjects with ON. Nonetheless, significantly different levels emerged when looking at the stress disorders scale of the PSQ. In closing, the hyper-activated lifestyle of people with orthorexia could favor psychophysiological hyperactivation that generates somatic complaints. In this direction, there is the 2021 study by Bartherls and colleagues [36], which showed that somatic symptoms, along with illness anxiety, are frequent in individuals with high levels of orthorexic eating behavior, in addition to healthy habits, amplification of perception of autonomic sensations, and hypochondriac concern for one’s health.

In summary, it seems that ON shares with OCD the presence of deficits in emotional regulation typical of syndromes with deficits in inhibitory control [48,49,50]. Furthermore, clinical psychophysiology studies attested to the inability of subjects with obsessive syndromes to deactivate the sympathetic activation induced by a stressor and to relax [51], finding confirmation in the present study. It highlighted the presence of a stress-related lifestyle, which predisposes to the onset of stress-related physical disorders, due to the absence of moments of relaxation and psychophysical recovery [51].

Considering the involvement of all parameters (cognitive, emotional, and behavioral) in ON, the need for a multidimensional assessment capable of describing all the characteristics of ON becomes clear. In this regard, it is underlined that the scientific literature, including the present study, has taken into account the cognitive and behavioral aspects of ON, neglecting the emotional–psychophysiological ones. Future studies will need to consider the various aspects of psychological suffering by including psychophysiological responses within the diagnostic evaluation. This approach would fit into the recently proposed Research Domain Criteria (RDoC) for the study of mental disorders, which aim to characterize psychopathology in terms of normal and abnormal biological and behavioral processes rather than as distinct symptom categories [19].

Although the absence of measurement of emotional regulation and psychophysiological arousal represents the main limitation of our research, there are others that further studies should overcome. First, the cross-sectional nature of this study cannot confirm the causality of the relationships between the observed variables. For instance, it was hypothesized that ON facilitates the implementation of repetitive behaviors and compulsions, such as augmented PA levels, and a lifestyle characterized by motor hyperactivity. In contrast, it is also possible that stress can accentuate obsessive thoughts as a strategy to control negative emotions. Additionally, ad hoc sampling may not represent the university student population because participants may have been driven by curiosity or motivation to learn more about certain aspects of themselves. In this regard, it is essential to consider the prevalence of ON that, even if consistent with the literature on university students (ranging from 7 to 83%) [19], is on the upper limit of the estimate. Nevertheless, the relationship between ON, hyperactivity, and the tendency to develop stress-related symptoms, such as somatization, would be investigated through more accurate measurements of various psychological variables. As already mentioned previously, these data could be associated with more objective ones (i.e., psychophysiological evaluation) as well as anthropometric data (i.e., weight, height, BMI) to determine the psychophysical impact of ON.

Despite the current limitations, our study shed light on a segment of ON assessment, proving its association with stress-related lifestyle and PA. Further studies are needed to shed light on the causality between these variables, but it is possible to hypothesize that the increase in PA represents a compulsion in response to the obsessive thoughts typical of individuals with orthorexia. Therefore, “obsessive” physical exercise may not generate the benefits generally reported by the literature [52] but, on the contrary, fuel the predisposition to develop stress-related disorders [53].

Lastly, the high prevalence of study participants with orthorexia is consistent with those of anxiety, depression [54], suicidal ideation [55], substance use, and eating disorders [56] attested by research studying the psychological symptoms and psychopathological disorders of university students. However, the worrying levels of emotional and behavioral disorders suggest the need to support the emotional regulation of this group of people and the implementation of functional and adaptive behaviors as well as promoting the ability to relax and lower the level of motor activity.

To conclude, ON involves different areas of a person’s life functioning and requires the involvement of different health professionals, such as trainers, nutritionists, and psychologists. To improve psychophysical well-being, psychologists should encourage the adoption of healthy behaviors and facilitate the implementation of strategies useful for managing emotions and facilitating cognitive flexibility, indirectly reducing excessive physical exercise. On the other hand, nutritionists and trainers have the delicate role of prescribing diets and training programs that emphasize the pleasure and gratification deriving from food and physical activity, accommodating the person’s preferences, and paying attention to behaviors and attitudes that may be a sign of underlying suffering.

## 5. Conclusions

Our study aimed to investigate the association between orthorexia, stress-related lifestyle, and PA. The findings described increased levels of motor and behavioral activity in people with ON, including higher levels of structured and unstructured PA. More specifically, people with orthorexia have a lifestyle characterized by hyper-responsibility associated with precision and difficulty in carving out time as well as motor–behavioral hyperactivity, which includes more hours of PA training. It has been hypothesized that the augmented motor and behavioral activity is a compensatory response to the obsessions typical of ON and reflects the inhibitory deficit and difficulties in the emotion regulation of obsessive–compulsive spectrum disorders certified by the scientific literature. These dimensions, in turn, could determine a greater predisposition to develop stress-related disorders. Further studies are necessary to investigate the relationship between these dimensions and support university students in implementing functional behaviors and regulating their negative emotions.

## Figures and Tables

**Table 1:** Comparisons of socio-demographic characteristics between the non-orthorexic and the orthorexic groups.

Variable	ORTO-15 Score > 40	ORTO-15 Score < 40	Total Sample (*n* = 166)	*t* or χ^2^	*p*
Age (years), M (SD)	24.21 (4.68)	24.73 (4.83)	24.62 (4.81)	*t* (165) = −0.60	0.27
Gender, *N* (%)				χ^2^ (1, *N* = 165) = 1.20	0.55
Male	10 (35.71%)	64 (46.38%)	74 (44.58%)		
Female	18 (64.29%)	74 (53.62%)	92 (55.42%)		
Marital status, *N* (%)				χ^2^ (1, *N* = 165) = 0.01	0.99
Unmarried	27 (96.43%)	133 (96.38%)	159 (95.78%)		
Married/cohabitant	1 (3.57%)	5 (3.62%)	7 (4.22%)		
Education level, *N* (%)				χ^2^ (1, *N* = 165) = 0.04	0.84
High school graduation	10 (35.71%)	52 (37.68%)	61 (36.75%)		
University degree	18 (64.29%)	86 (62.32%)	102 (63.25%)		

**Table 2:** Comparisons of behavioral characteristics between the non-orthorexic and the orthorexic groups.

	ORTO-15 Score > 40		ORTO-15 Score < 40		Total Sample (*n* = 166)		*t* (165)	*p*	Cohen’s D
	M	SD	M	SD	M	SD			
P Stress Questionnaire									
Sense of responsibility	5.18	3.07	6.54	3.34	6.31	3.33	−1.99	0.02	−0.42
Vigor	2.68	2.47	3.28	2.34	3.18	2.36	−1.24	0.11	−0.26
Stress disorder	2.96	1.68	3.35	1.66	3.28	1.70	−1.38	0.05	−0.29
Precision and punctuality	3.53	1.74	4.32	1.98	3.66	1.96	1.99	0.03	0.41
Spare time	1.25	1.65	2.04	2.00	1.91	1.96	−1.97	0.03	−0.41
Hyperactivity	4.75	1.80	5.28	1.48	5.19	1.54	−1.68	0.04	−0.35
Total	23.59	6.90	27.18	6.68	26.44	7.55	−2.53	0.005	−0.58

**Table 3:** Comparisons of the physical exercise characteristics between the non-orthorexic and the orthorexic groups.

Variable	ORTO-15 Score > 40	ORTO-15 Score < 40	Total Sample (*n* = 166)	*t* or χ^2^	*p*	Cohen’s D
Structured physical activity, *N* (%)				χ^2^ (1, *N* = 165) = 1.14	0.29	
Yes	20 (71.43%)	111 (80.43%)	131 (78.92%)			
No	8 (28.57%)	27 (19.57%)	35 (25.36%)			
Hours of structured physical activity, M (SD)						
Daily	1.19 (1.11)	1.61 (1.21)	1.54 (1.20)	*t* (165) = −1.68	0.05	−0.35
Weekly	2.92 (3.07)	4.18 (3.04)	3.97 (3.07)	*t* (165) = −1.91	0.03	−0.41
Monthly	11.69 (12.29)	16.70 (12.18)	15.82 (12.30)	*t* (165) = −1.91	0.03	−0.41
Unstructured physical activity, M (SD)						
Using stairs vs. elevator	62.69 (28.50)	64.34 (27.69)	64.07 (27.74)	*t* (165) = −0.28	0.39	0.06
Move with bike or walk vs. transport	80.77 (14.12)	74.63 (23.88)	75.62 (22.68)	*t* (165) = 1.27	0.04	0.27
Walk alone/in company vs. sedentary activities	61.92 (22.09)	61.25 (26.40)	61.36 (25.70)	*t* (165) = 0.12	0.45	0.03
Go to a friend vs. call him	76.15 (21.55)	76.74 (21.61)	76.65 (21.53)	*t* (165) = −0.13	0.45	−0.03
Swim or play on the beach vs. lie down and sunbathe	68.85 (27.62)	75.33 (23.75)	74.29 (24.44)	*t* (165) = −1.24	0.11	−0.27
Carrying out physical activity even if tired	42.31 (29.57)	55.41 (31.31)	53.29 (31.32)	*t* (165) = −1.97	0.02	−0.42
Talk/go for a walk vs. eat after arguing	66.92 (29.50)	68.59 (27.13)	68.32 (27.44)	*t* (165) = −0.28	0.39	−0.06

## Data Availability

The data presented in this study are available upon reasonable request from the corresponding author due to privacy.
